# *Pennisetum glaucum* (L.) Oral Supplementation Mitigates Multi-Organic Dysfunction Associated with Carcinogenesis in HPV16-Transgenic Mice

**DOI:** 10.3390/cimb47100858

**Published:** 2025-10-17

**Authors:** Paula A. Oliveira, Latifa Hajri, Armando V. Pinto Moreno, Carlos E. Dias Santos, Haissa O. Brito, Margarida M. S. M. Bastos, Rui Medeiros, Soumaya Ghodbane, Mohamed Ammari, Rui M. Gil da Costa, Ana I. Faustino-Rocha

**Affiliations:** 1 Department of Veterinary Sciences, University of Trás-os-Montes and Alto Douro (UTAD), 5000-801 Vila Real, Portugal; 2 Centre for the Research and Technology of Agro-Environmental and Biological Sciences (CITAB), Institute for Innovation, Capacity Building and Sustainability of Agri-Food Production (Inov4Agro), University of Trás-os-Montes and Alto Douro (UTAD), 5000-801 Vila Real, Portugal; 3 Laboratory of Integrative Physiology, Faculty of Sciences of Bizerte, University of Carthage, Jarzouna 7021, Tunisia; 4 Research Center for Experimental and Clinical Physiology and Pharmacology (NEC), Federal University of Maranhão (UFMA), São Luís 65080-805, Brazil; 5 Laboratory for Process Engineering, Environment, Biotechnology and Energy (LEPABE), Associate Laboratory in Chemical Engineering (ALiCE), Faculty of Engineering, University of Porto, 4200-465 Porto, Portugal; 6 Molecular Oncology and Viral Pathology Group, Research Center of IPO-Porto (CI-IPOP) & RISE@CI-IPOP (Health Research Network), Portuguese Oncology Institute of Porto (IPO-Porto)/Porto Comprehensive Cancer Center Raquel Seruca (Porto.CCC), 4200-072 Porto, Portugal; 7 ICBAS—Instituto de Ciências Biomédicas Abel Salazar, University of Porto, 4050-313 Porto, Portugal; 8 Faculty of Medicine, University of Porto (FMUP), 4200-072 Porto, Portugal; 9 Faculty of Health Sciences, Fernando Pessoa University, 4200-150 Porto, Portugal; 10 Research Department, Portuguese League Against Cancer (NRNorte), 4200-172 Porto, Portugal; 11 Higher Institute of Applied Biological Sciences of Tunis, University of Tunis El Manar, Tunis 7021, Tunisia; 12 Department of Pathology, State University of Maranhão, São Luís 65055-310, Brazil; 13 University of Évora, School of Sciences and Technology, Department of Zootechnics, 7004-516 Évora, Portugal; 14 University of Évora, Comprehensive Health Research Center (CHRC), 7004-516 Évora, Portugal

**Keywords:** cancer, paraneoplastic syndrome, functional food

## Abstract

Cancers induced by human papillomavirus are often associated with systemic inflammation and cachexia. This study aimed to determine the interference of *Pennisetum glaucum* oral supplementation over multi-organic dysfunction in HPV16-transgenic mice. The experimental groups included (1) wildtype (WT) mice with standard diet, (2) WT mice with 36% *Pennisetum*, (3) transgenic mice with standard diet, (4) transgenic mice with 29% *Pennisetum*, and (5) transgenic mice with 36% *Pennisetum*. During the 4-week experimental protocol, body weight, food and water intake, and humane endpoints were recorded. At sacrifice, blood and tissue samples were collected for analysis. Oral supplementation with millet was shown to be safe and well tolerated by both WT and transgenic mice, with no adverse effects on behavior, food or water intake, or general animal welfare. In HPV16-transgenic animals, millet supplementation was associated with an improved health status, reduced serum glucose levels, enhanced antioxidant responses, and a notable reduction in the severity of HPV-induced skin and organ lesions. Overall, *Pennisetum glaucum* was safe under these experimental conditions and is a promising functional food for patients suffering from systemic paraneoplastic syndromes. Longer exposure periods and doses should be evaluated experimentally before proceeding to clinical trials of *Pennisetum*-containing diets.

## 1. Introduction

Human papillomavirus (HPV) belongs to the Papillomaviridae family. It is characterized as a small, double-stranded, circular, and enveloped virus with a diameter of 50 to 55 nm [1]. Its genome contains three regions, one called the late region (L—late) in which the L1 and L2 genes are present, responsible for coding the viral protein capsules. The early region (E—early) is made up of the E1 and E2 genes, which are involved in viral replication and transcription control, the E4 gene, which is responsible for viral maturation and modification of the extracellular matrix, and the E5, E6, and E7 genes, which interfere with key cell functions and may drive malignant transformation. Finally, the long control region (LCR) between the L and E regions includes nuclear and viral transcription factors [2]. The infection process begins when viral particles come into contact with the basal cells of the host’s epithelium. When basal cells divide, the HPV DNA is distributed among daughter cells and the infection persists [3]. However, only when the infected basal cell fully differentiates and moves to the upper layers of the epithelium is it possible to synthesize new infectious viral particles. At this point, the E6 and E7 proteins prevent the loss of the nuclear machinery and stimulate the replication of the infected epithelial cells, potentially resulting in cell transformation [2]. There are more than 100 types of HPV, and all infect epithelial tissues. High-risk oncogenic HPV infects the anal, oropharyngeal, and genital tracts and induces the development of cancer in these regions [1]. Other types of HPV, classified as low-risk, mostly induce the development of benign hyperproliferative lesions or genital warts [4]. Currently, there are 12 high-risk HPV types related to malignant neoplasms (HPV-16, 18, 31, 33, 35, 39, 45, 51, 52, 56, 58, and 59) [5,6]. HPVs are grouped into the following genera: alphapapilomavirus, betapapilomavirus, gamapapilomavirus, mupapilomavirus, and nupapilomavirus [7]. Oncogenic HPV is strongly associated with cervical cancer, and its prevalence among patients with head and neck cancers, particularly oropharyngeal cancers, is increasing at an alarming rate. In developed Western nations, HPV is responsible for round 90% of all oropharyngeal cancer cases, and the proportion of HPV-related oropharyngeal cancers is also rising in developing countries [8]. Although vaccines and screening technologies are highly effective in preventing HPV-induced cancers, the global burden of these cancers remains disproportionately high in low- and middle-income countries. Eliminating cervical cancer and other HPV-related diseases is a scientific and a moral imperative, requiring global collaboration and local action [9].

Over the years, there have been several important milestones that have advanced the study of the complexity and development of cancer, including insights into its molecular mechanisms. However, molecular analysis of clinical samples is often difficult, e.g., when it is necessary to obtain several biopsies at different stages of the tumor. For this reason, experimental murine cancer models have been used, as they allow for a better understanding of tumors on their in vivo environments, allowing for the better assessment of new diagnostic methods and therapies [10]. The K14-HPV16 model was developed from FBV/n mice to study the pathophysiology of HPV-induced alterations in these animals. In this murine model, the human promoter of the cytokeratin 14 gene *Krt14* directs the expression of the HPV16 early genes to the basal layer of squamous epithelia, leading to the spontaneous development of lesions characteristic of infection by this virus in humans [11,12,13]. In this model, epithelial carcinogenesis is accompanied by systemic effects mimicking chronic inflammation and cachexia, as observed in human cancer patients [14,15].

A *Pennisetum glaucum* (L.), also known as pearl millet, is an annual plant that grows very quickly and can mostly be found in tropical areas. It adapts to a wide range of environments and climates. The fact that it can be easily grown anywhere makes it a very attractive plant species for producers, as it can adapt to dry climates, high temperatures, and different types of soil. This plant is considered a staple food in Africa and Asia and has a high nutritional value due to its high fat, protein, zinc, and iron content [16]. Several functional properties have been described for *Pennisetum glaucum*, including the prevention of cancer and cardiovascular diseases, the lowering of blood pressure and cholesterol, the reduction of the glycemic index, immunomodulatory and anti-inflammatory effects, the delaying of the feeling of an empty stomach, weight management, and the mitigation of the risk of chronic diseases such as diabetes. These properties align with contemporary health concerns [17,18,19]. *Pennisetum glaucum* also has antioxidant, antimicrobial, antidiabetic, anti-aging, and antiallergic effects [20].

We hypothesize that a diet containing *Pennisetum glaucum* will be able to counter, at least partially, chronic inflammation and involuntary weight loss associated with cancer. Accordingly, the present study aimed to test the ability of oral supplementation with *Pennisetum glaucum* to mitigate the systemic effects of epithelial carcinogenesis driven by HPV16 on a genetically modified K14-HPV16 animal model.

## 2. Materials and Methods

### 2.1. Ethical Approval

All biosecurity standards for studies using animal models were respected (European Directive 2010/60/EU and National Decree-Law 113/2013). This work was approved by the Portuguese Veterinary Directorate (approval no. 014139) and the University of Trás-os-Montes and Alto Douro (UTAD) Ethics Committee for Animal Welfare (approval no. 852-e-CITAB-2020_A_1-e-CITAB-2021, 16 October 2021).

### 2.2. Animals

Twenty-five FVB/n male Mus musculus of the FVB/n strain, aged 10–20 weeks, were employed, including 10 wildtype (HPV−, WT) and 15 transgenic (HPV+) mice. This genetically modified strain was donated by Drs. Jeffrey Arbeit and Douglas Hanahan (University of California) through the National Cancer Institute Mouse Repository [21], and the animals were genotyped as previously described by Paiva et al. [22]. The animals were housed at the UTAD animal facilities in polycarbonate cages, with corncob for bedding, under controlled conditions: light/dark cycle (12 h light/12 h dark), humidity (50 ± 10%) temperature (23 ± 2 °C), and air system filtration (10–20 ventilations/hour). Control animals were fed with a standard laboratory diet (4RF21^®^, Mucedola, Settimo, Italy), while the treated animals were fed with a standard laboratory diet supplemented with either 29% or 36% *Pennisetum glaucum*. Food and tap water were provided ad libitum.

### 2.3. Pennisetum Glaucum

*Pennisetum glaucum* was sourced from Bizerte (Tunisia) and kindly collected and donated by local farmers. The botanical identification of the plant was carried out at the University of Pharmacy in Monastir, Tunisia. The phytochemical characterization of the *Pearl millet* extract is described in detail in the Supplementary File. The seeds were ground and incorporated into the standard diet at concentrations of 29% and 36%. Both the seeds and the standard feed were ground in a Bimby blender (Vorwerk, Wuppertal, Germany) and then mixed at the respective concentrations. Once the new diets had been made with the desired concentrations, the pellets were dried in a high-precision incubator (HotCold260, Astori Tecnica, Poncarale, Italy) at 40 °C for two days. Once the cooking and drying process was complete, the food was stored in a cool, dry place.

### 2.4. Experimental Design

WT and HPV+ mice were randomly ascribed to five experimental groups (n = 5/group) as follows: group 1—WT mice with standard diet; group 2—WT mice supplemented with 36% *Pennisetum glaucum*; group 3—HPV+ with standard diet; group 4—HPV+ mice supplemented with 29% *Pennisetum glaucum*; group 5—HPV+ mice supplemented with 36% *Pennisetum glaucum*. The protocol lasted four weeks. During the experimental protocol, the animals were observed daily to check their general health status. The animal welfare was evaluated, once a week, using a table of humane endpoints previously established by Oliveira et al. [23]. We evaluated several aspects, including general appearance (body condition, body weight, food and water intake, posture, coat and grooming, mucosal, eyes, ears, and whiskers), behavior (response to external stimuli), mental status, body temperature, respiratory rate, presence of papillomas, and feces appearance. A score from zero to four was assigned to each parameter. A score of four or higher was indicative for animals’ euthanasia.

### 2.5. Body Weight, Food and Water Consumption, and Lee Index

The body weight (BW) of the animals and the food and water weights were recorded on a weekly basis to estimate BW variations and food and water consumption. At the end of the protocol, ponderal weight gain was calculated by subtracting the initial BW from the final BW, dividing by the initial BW, and multiplying by 100 [24,25]. The Lee index was calculated as the cube root of the final BW divided by the naso-anal length of the animal multiplied by 100 [26]. Body mass index was calculated from the ratio of the final BW to the naso-anal length squared [25,27].

### 2.6. Animals Sacrifice and Necropsy

At the end of the four-week experimental period, the animals were fasted for 12 h before being euthanized via intraperitoneal injection of an overdose of ketamine (75 mg/kg, Imalgene 1000, Merial SAS, Lyon, France) and xylazine (10 mg/kg, Rompun 2%, Bayer Healthcare SA, Kiel, Germany), followed by exsanguination by cardiac puncture, as indicated by the Federation for Laboratory Animal Science Associations (FELASA). A complete necropsy was performed, and organ samples (heart, kidneys, lungs, spleen, liver, ear skin, chest skin) were collected and immersed in 10% neutral buffered formalin for 24 h.

### 2.7. Blood Sample Collection and Analysis

Blood was collected directly from the heart into tubes with ethylene diamine tetraacetic acid (EDTA) for hematological analysis, which was then carried out on equipment IDEXX ProCyte Dx Hematology system (Westbrook, ME, USA) and included erythrocytes, hematocrit, hemoglobin, mean cell volume (MCV), mean corpuscular hemoglobin (MCH), mean corpuscular hemoglobin concentration (MCHC), reticulocyte count, leukocyte count, neutrophil count, lymphocyte count, monocyte count, eosinophil count, basophil count, platelet count, mean platelet volume (MPV), platelet distribution width (PDW), and total protein. The glucose levels were determined using a GlucoMen Areo 2K glucometer (Menarini Diagnostics, Paço de Arcos, Portugal).

### 2.8. Oxidative Stress Analysis

The activity of superoxide dismutase (SOD), catalase (CAT), glutathione peroxidase (GPx), glutathione reductase (GR), glutathione S-transferase (GST), glutathione in the reduced state (GSH), glutathione in the oxidized state (GSSG), oxygen reactive species (ROS), thiobarbituric acid reactive substances (TBARS), and carbonyls were evaluated as markers of oxidative stress in the liver and kidney. Each liver lobe sample and a portion of the kidney was thawed and homogenized using a Potter homogenizer in cold phosphate-buffered saline (100 mM-EDTA 1 mM, pH 7.4). The samples were homogenized in an ice bath using an ultrasound processor (4 × 20 s, intermittent 20 s). After homogenization, the samples were centrifuged at 2000× *g* for 10 min. The resulting supernatant underwent a second centrifugation at 1200× *g* for 10 min, and the last supernatant was collected in an Eppendorf tube for further analysis. The activity results were normalized to the protein content of the samples, which was determined using a BioTekTissGen5TM (Powerwave XS2, BioTek Instruments, Inc. Winooski, VT, USA) based on the absorbance measurements at 280 nm.

### 2.9. Histological Analysis

Tissue samples fixed in 10% neutral buffered formalin were paraffin-embedded and sectioned at 4 µm for hematoxylin and eosin staining. Histological slides were examined under a light microscope (O500S, Opticam, Montreal, QC, Canada), and photographed using a OPT12MP camera and OPTHD software (version 1.7). Ear and chest skin lesions were classified as epidermal hyperplasia, epidermal dysplasia, and SCC, as previously described [14]. Hepatic lesions were classified as grade I and grade II hepatitis, while splenic lesions were classified as white pulp hyperplasia, as previously described [14].

### 2.10. Statistical Analysis

The statistical analysis was carried out using Statistical Package for Social Sciences version 30 (IBM, Chicago, IL, USA) and GraphPad Prism version 10.1.1 (GraphPad Software, Boston, MA, USA). Continuous data were compared among groups using analysis of variance (ANOVA) followed by post hoc Tukey’s test for multiple comparisons. Histological data were examined using the Chi-squared test. Data are presented as mean ± standard error (S.E.). *p* values lower than 0.05 were considered statistically significant.

## 3. Results

### 3.1. General Results

The phytochemical characterization of the *Pearl millet* extract is available in the Supplementary File (Figures S1–S3) [14,17,20,21,22]. During the experiment, the animals showed no signs of behavioral changes, and no mortality was observed. Throughout the protocol, the humane endpoints were analyzed once a week for each animal. Both wildtype groups (groups 1 and 2) showed scores of 0.00 in weeks 1 and 4. Group 3 (HPV+, standard diet) had the highest score in the last week of the experiment. Statistically significant differences were not found among the groups (*p* > 0.05) (Table 1).

### 3.2. Food and Water Consumption

**Figure 1:**
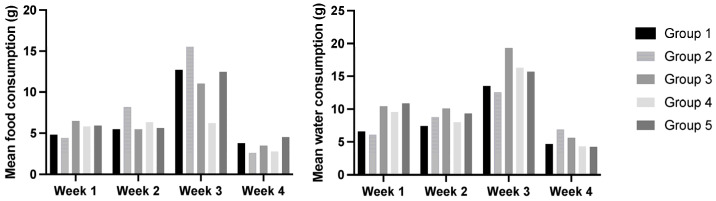
Mean food and water consumption per animal (g) in each experimental group in all weeks of the experiment. Data are presented as mean ± standard error (S.E.). Statistically significant differences were not found (*p* > 0.05). Groups: group 1—WT mice with standard diet; group 2—WT mice supplemented with 36% *Pennisetum glaucum*; group 3—HPV+ with standard diet; group 4—HPV+ mice supplemented with 29% *Pennisetum glaucum*; group 5—HPV+ mice supplemented with 36% *Pennisetum glaucum*.

No statistically significant changes were observed in the food and water consumption of any of the groups over the four weeks of the experiment (*p* > 0.05). For all groups, the highest food and water consumption was observed at week 3 of the experiment, which decreased between the first and the last week of the experiment (*p* > 0.05) (Figure 1).

### 3.3. Body Weight and Murinometric Variables

**Figure 2:**
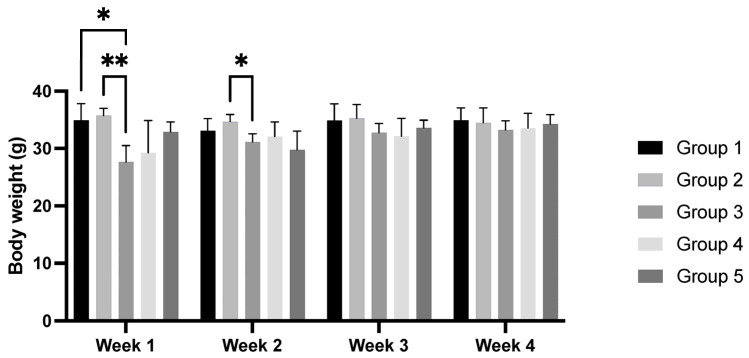
Mean animal body weight (g) in each experimental group in all weeks of the experiment. Data are presented as mean ± standard error (S.E.). * *p* < 0.05; ** *p* < 0.01. Groups: group 1—WT mice with standard diet; group 2—WT mice supplemented with 36% *Pennisetum glaucum*; group 3—HPV+ with standard diet; group 4—HPV+ mice supplemented with 29% *Pennisetum glaucum*; group 5—HPV+ mice supplemented with 36% *Pennisetum glaucum*.

In general, the HPV+ groups (groups 3, 4, and 5) had a lower body weight than the WT groups (groups 1 and 2). In the second week of the experiment, the mean body weight of group 3 (HPV+, standard diet) was lower when compared with that of group 2 (WT, 36% *Pennisetum glaucum*) (*p* < 0.05). No changes in body weight were observed in the last two weeks of the experiment (*p* > 0.05) (Figure 2).

Statistically significant differences were not found in the body mass index, naso-anal length, naso-caudal length, abdominal perimeter, or Lee index among the groups (Table 2). The animals from the HPV groups (groups 3, 4, and 5) presented a positive ponderal weight gain, while group 2 (WT, 36% *Pennisetum glaucum*) presented weight loss. Among the HPV+ groups, group 5 (HPV+, 36% *Pennisetum glaucum*) had the lowest ponderal weight gain. A statistically significant difference was observed between groups 2 and 3 (*p* < 0.05) (Table 2).

### 3.4. Organs Weight

At the end of the experimental work, the animals were sacrificed, and their internal organs were collected and weighed. Table 3 shows the absolute and relative weights of the organs, as well as the respective standard errors for the different groups under study. The absolute and relative weights of the heart, lungs, spleen, and left kidney were similar among the groups (*p* > 0.05). The liver relative weight was highest in groups 3 (HPV+, standard diet) and 5 (HPV+, 36% *Pennisetum glaucum*), when compared with group 2 (WT, 36% *Pennisetum glaucum*) (*p* < 0.05). The right kidney absolute weight was highest in group 1 (WT, standard diet) when compared with groups 3 (HPV+, standard diet) and 4 (HPV+, 29% *Pennisetum glaucum*) (*p* < 0.05). The right kidney absolute weight was highest in group 5 (HPV+, 36% *Pennisetum glaucum*) when compared with groups 3 (HPV+, standard diet) and 4 (HPV+, 29% *Pennisetum glaucum*) (*p* < 0.05).

### 3.5. Hematological Parameters

After sacrifice, a hematological study was carried out. Table 4 shows the mean and standard error results for the hematological parameters, serum total proteins, and glucose. There were no statistically significant differences in the hematological parameters among the groups (*p* > 0.05). The serum glucose levels were higher in groups 3 (HPV+, standard diet) and 4 (HPV+, 29% *Pennisetum glaucum*) when compared with groups 1 (WT, standard diet), 2 (WT, 36% *Pennisetum glaucum*), and 5 (HPV+, 36% *Pennisetum glaucum*) (*p* < 0.05). The concentration of glucose was lower in group 5 when compared with other HPV+ groups (groups 3 and 4), suggesting that the higher concentration of *Pennisetum glaucum*, the lower the concentration of glucose (*p* < 0.05).

### 3.6. Oxidative Stress Analysis

**Figure 3:**
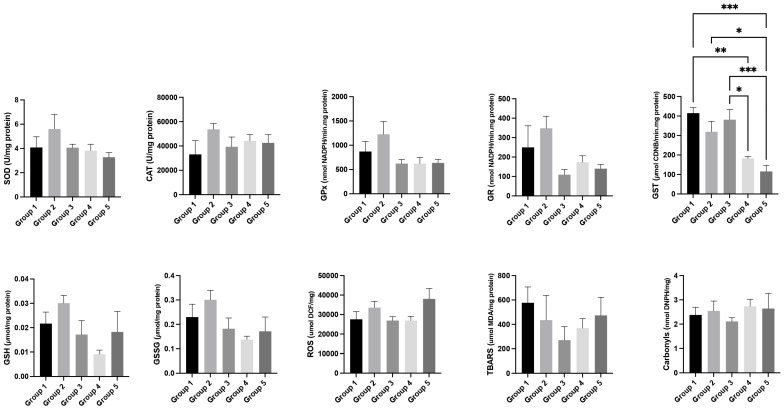
Oxidative stress parameters in the liver for each experimental group. Data are presented as mean ± standard error (S.E.). * *p* < 0.05; ** *p* < 0.01; *** *p* < 0.001. Groups: group 1—WT mice with standard diet; group 2—WT mice supplemented with 36% *Pennisetum glaucum*; group 3—HPV+ with standard diet; group 4—HPV+ mice supplemented with 29% *Pennisetum glaucum*; group 5—HPV+ mice supplemented with 36% *Pennisetum glaucum*.

Regarding liver oxidative stress, in a general way, the levels of SOD, CAT, GPx, GR, GST, GSH, GSSG, ROS, TBARS, and carbonyls were lower in the HPV+ groups (groups 3, 4, and 5) when compared with the WT groups (groups 1 and 2) (Figure 3). It is worth noting that the lowest levels of GST were observed in the HPV+ group treated with the highest concentration of *Pennisetum glaucum* (group 5; HPV+, 36% *Pennisetum glaucum*) (*p* < 0.05) (Figure 3).

**Figure 4:**
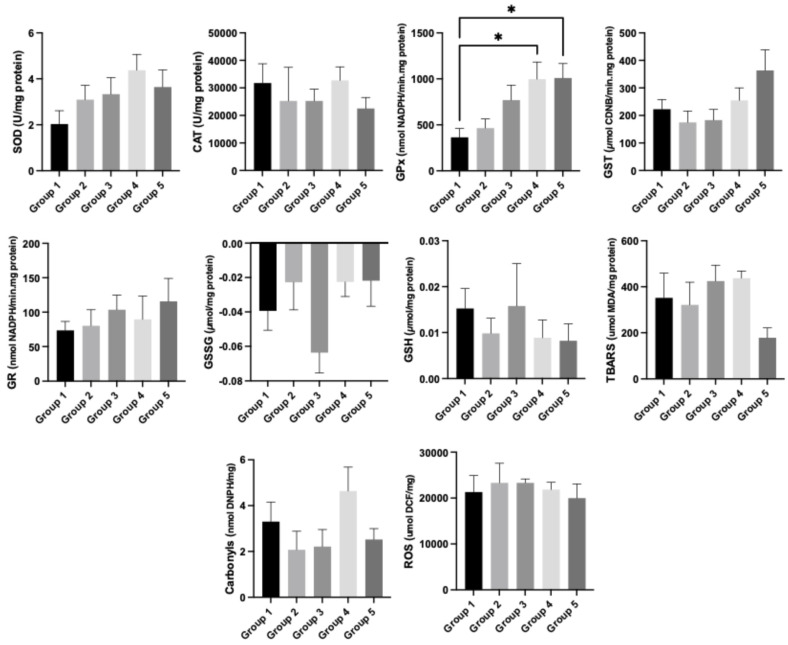
Oxidative stress parameters in the kidney for each experimental group. Data are presented as mean ± standard error (S.E.). * *p* < 0.05. Groups: group 1—WT mice with standard diet; group 2—WT mice supplemented with 36% *Pennisetum glaucum*; group 3—HPV+ with standard diet; group 4—HPV+ mice supplemented with 29% *Pennisetum glaucum*; group 5—HPV+ mice supplemented with 36% *Pennisetum glaucum*.

Regarding kidney oxidative stress, in a general way, the levels of stress markers were higher in the HPV+ groups (groups 3, 4 and 5) compared to the WT groups (groups 1 and 2). The levels of GPx were higher in groups 4 (HPV+, 29% *Pennisetum glaucum*) and 5 (HPV+, 36% *Pennisetum glaucum*), when compared with group 1 (WT, standard diet) (*p* < 0.05; Figure 4).

### 3.7. Epithelial Carcinogenesis

**Figure 5:**
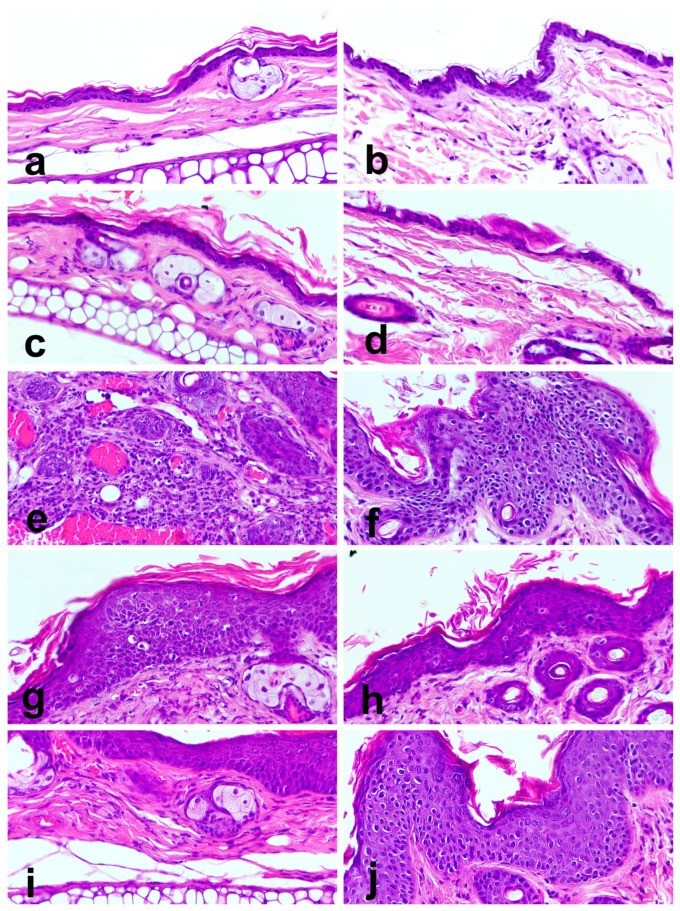
Epithelial carcinogenesis driven by HPV16 oncogenes in transgenic mouse skin, stained with hematoxylin and eosin, 400× magnification. Panels (**a**,**b**) show ear and chest skin, respectively, from wildtype (WT) mice receiving standard diet (group 1). Note normal skin histology. Panels (**c**,**d**) show ear and chest skin, respectively, from WT mice receiving 36% *Pennisetum*-supplemented diet (group 2). Note normal skin histology. Panels (**e**,**f**) show ear and chest skin, respectively, from HPV+ mice receiving standard diet (group 3). Note a squamous cell carcinoma (SCC) in panel (**e**) and epidermal dysplasia in panel (**f**). Panel (**g**,**h**) show ear and chest skin, respectively, from HPV+ mice receiving 29% *Pennisetum*-supplemented diet (group 4). Note epidermal dysplasia in both panels. Panels (**i**,**j**) show ear and chest skin, respectively, from HPV+ mice receiving 36% *Pennisetum*-supplemented diet (group 5). Note epidermal hyperplasia in both panels.

All WT animals (groups 1 and 2) showed normal skin histology, regardless of eating the standard diet or the *Pennisetum*-supplemented diet (Table 5, Figure 5a–d). Conversely, HPV+ animals showed lesions ranging between epidermal hyperplasia and SCC (Table 5, Figure 5e–j). Hyperplastic lesions showed an increased number of epidermal layers, ranging between 4 and 10 (Figure 5i,j). Normal epidermal differentiation was retained and accompanied by hyperkeratosis. Epidermal cells showed mild to moderate nuclear pleomorphism, and mitotic figures were restricted to basal or parabasal layers. In dysplastic lesions, most epidermal cell layers were replaced with basaloid cells, and there was abrupt keratinization (Figure 5f–h). Cells showed intensely pleomorphic nuclei and mitotic figures in the suprabasal layers, indicating severe disruption of normal epidermal differentiation. Squamous cell carcinomas showed cytological features similar to epidermal dysplasia, but nests of neoplastic cells invaded the underlying dermis (Figure 5e). Dermal leukocytic infiltration accompanied the development of lesions, growing in intensity from hyperplastic through dysplastic to SCC lesions.

### 3.8. Organ Toxicity

**Figure 6:**
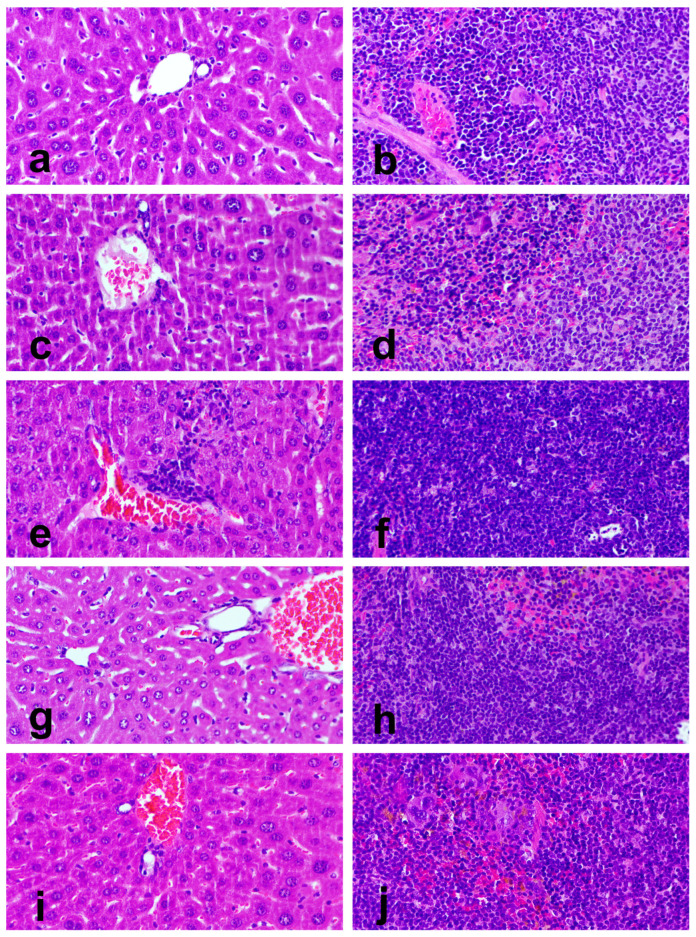
Hepatic and splenic histology in experimental mice, stained with hematoxylin and eosin, 400× magnification. Panels (**a**,**b**) show liver and splenic histology, respectively, from WT mice receiving standard diet (group 1). Note normal histology. Panels (**c**,**d**) show liver and splenic histology, respectively, from WT mice receiving 36% *Pennisetum*-supplemented diet (group 2). Note normal histology. Panels (**e**,**f**) show liver and splenic histology, respectively, from HPV+ mice receiving standard diet (group 3). Note a grade II hepatitis in panel (**e**) and splenic white pulp hyperplasia in panel (**f**). Panel (**g**,**h**) show liver and splenic histology, respectively, from HPV+ mice receiving 29% *Pennisetum*-supplemented diet (group 4). Note normal histology in both panels. Panels (**i**,**j**) show liver and splenic histology, respectively, from HPV+ mice receiving 36% *Pennisetum*-supplemented diet (group 5). Note normal histology in both panels.

No lesions were observed in the lung, heart, or kidneys. On histological analysis, the organs of WT mice showed normal tissue architecture, irrespective of receiving the standard diet or the *Pennisetum*-supplemented diet (Figure 6a–d). In contrast, HPV+ mice showed hepatitis and splenic white pulp hyperplasia at varying rates between groups (Table 6, Figure 6e–j). Grade I hepatitis consisted of Küpfer cell hyperplasia and occasional microabscesses, while grade II hepatitis showed severe periportal leukocytic infiltration, including macrophages, lymphocytes, and neutrophils. Splenic white pulp hyperplasia was characterized by enlarger periarteriolar lymphoid follicles that compress and partially replace the splenic red pulp.

## 4. Discussion

The present study aimed to evaluate the interference of oral supplementation with *Pennisetum glaucum* on the genetically modified K14-HPV16 animal model. *Pennisetum glaucum*, commonly known as millet, is a whole grain rich in protein, fiber, minerals, and bioactive compounds that are endowed with functional activities, acting as hypoglycemic, antioxidant, and anti-inflammatory food [19,28,29]. Considering that chemical and viral agents may be involved simultaneously in carcinogenesis, we used a transgenic animal model for HPV16 and incorporated two different concentrations of *Pennisetum glaucum* seeds in the food to study its interference with the physiological variables and the development of HPV16-induced lesions. We also determined and analyzed the hematological parameters and oxidative stress of each animal at the end of the trial. It is known that some of the signs of toxicity associated with the administration of compounds of natural origin are animal mortality and behavioral changes [30]. The use of humane endpoints and weekly scoring is standard practice to ensure animal welfare and identify suffering early. No behavioral changes or mortality were registered during the experiment. The humane endpoints monitored during the study suggest that the general condition of the animals was maintained, and it was not necessary to sacrifice any animal before the planned end date of the experimental work, demonstrating that *Pennisetum glaucum* does not compromise animal welfare [31]. Among the transgenic animals (HPV+ animals), the groups supplemented with *Pennisetum glaucum* (group 4 (HPV+ supplemented with 29% *Pennisetum glaucum*) and group 5 (HPV+ supplemented with 36% *Pennisetum glaucum*)) presented a lower score for humane endpoints when compared with the transgenic animals fed the standard diet (group 3 (HPV+, standard diet)), suggesting that oral supplementation with *Pennisetum glaucum* improved the animals’ health status. During the experimental trial, the food and water consumption were recorded. No changes were observed in the pattern of water and food consumption over the trial in either wildtype or transgenic mice, suggesting that millet has a pleasant taste for mice. In work with K14-HPV16 mice, Gil da Costa and his collaborators [14] found that water consumption was higher in transgenic animals, which was not the case in this trial. This lack of significant variation in consumption supports the animal welfare status recorded by humane endpoint assessment. Despite the lack of differences in food and water consumption throughout the experiment, in the last week of the trial, group 5 (HPV+ mice supplemented with 36% *Pennisetum glaucum*) was the transgenic group with the highest body weight, which can be related to the *Pennisetum glaucum* supplementation [28]. The murinometric parameters of naso-anal length, naso-caudal length, and abdominal perimeter were similar among the groups, suggesting that neither the transgenesis nor *Pennisetum glaucum* supplementation influenced these morphometric variables. However, the ponderal weight gain was negative in group 2 (WT mice supplemented with 36% *Pennisetum glaucum*), suggesting that supplementation negatively affects body weight variation in WT animals. In contrast, Magalhães et al. [32] observed a decrease in these parameters in male Wistar rats (21 days old). The liver relative weight was higher in groups 3 (HPV+ mice, supplemented with standard diet) and 5 (HPV+ mice, supplemented with 36% *Pennisetum glaucum*) than in group 2 (WT mice, supplemented with 36% *Pennisetum glaucum*), suggesting that diet can affect liver weight in non-transgenic animals. Similar results were described by our team in 2017, in which the transgenic animals tended to exhibit a significant increase in liver and spleen mass due to the inflammatory processes following transgenesis [14]. Similarly, the variations in the right kidney absolute and relative weights suggest that diet supplementation and transgenesis also affect this organ’s weight. Statistically significant differences were not found in most hematological parameters among the groups, suggesting no interference of *Pennisetum* supplementation in these parameters. Among the transgenic animals, the highest serum glucose levels were observed in group 3 (HPV+ mice, with standard diet), while the lowest values were observed in group 5 (HPV+ mice, supplemented with 36% *Pennisetum glaucum*), suggesting that the greater the amount of millet, the lower the glucose in transgenic animals. This may be explained by the very high amylase activity of millet and because the main sugars in millet are maltose and D-ribose, which are low in fructose and glucose [33]. Surprisingly, inconsistent results were found regarding oxidative stress markers in liver and kidney tissues. Glutathione S-transferases (GSTs) are a superfamily of enzymes that metabolize xenobiotic substances, playing a key role in detoxifying harmful electrophilic compounds and mediating responses to oxidative stress. GST activity levels can fluctuate, often increasing to counteract the damaging effects of excessive reactive oxygen species (ROS) [34]. In this study, liver analysis revealed that transgenic groups exhibited the lowest GST levels, which decreased further with increasing supplementation of *Pennisetum glaucum*. This trend suggests a reduction in ROS and highlights the potential beneficial effects of *P. glaucum* supplementation in K14-HPV16 transgenic mice at the liver level. In mammals, GPX works alongside superoxide dismutase SOD and catalase CAT as part of the enzymatic antioxidant system that reduces ROS and limits their toxicity, and influencing nearly all cellular processes [35]. A study conducted in 2014 demonstrated that human papillomavirus type 16 (HPV16) increased ROS levels in both HPV-positive and HPV-negative cells [36]. In the present study, a significant increase in GPX levels was observed in the kidneys of transgenic animals supplemented with Pennisetum glaucum, suggesting that this supplementation had no substantial effect on reducing ROS levels at the renal level. Concerning skin histopathology, the WT animals (groups 1 and 2) presented no lesions in ear or chest skin, as expected. Conversely, all animals from the HPV+ groups (groups 3, 4 and 5) presented alterations in ear and chest skin. The animals from group 3 (HPV+ mice, with standard diet) were those with high-grade lesions, suggesting that supplementation with millet had a positive effect. It is worth noting that group 3 was the only one presenting a SCC. Analysis of liver and kidney is critical to determine the impact of laboratory animal exposure to different compounds [36]. WT animals (groups 1 and 2) presented no lesions in the liver or spleen. Millet supplementation reduced hepatitis in the transgenic supplemented animals. A similar effect was observed in spleen histopathological analysis in group 5 (HPV+ mice, supplemented with 36% *Pennisetum glaucum*), with animals presenting no alterations.

## 5. Conclusions

This study aimed to evaluate the impact of millet (*Pennisetum glaucum*) oral supplementation on the genetically modified K14-HPV16 animal model, examining the interaction between diet and the evolution of the animals’ general condition and the development of HPV-induced lesions over a four-week feeding period. Oral supplementation with millet was shown to be safe and well tolerated by both WT and transgenic mice, with no adverse effects on behavior, food or water intake, or general animal welfare. In HPV16-transgenic animals, millet supplementation was associated with an improved health status, reduced serum glucose levels, enhanced antioxidant responses, and a notable reduction in the severity of HPV-induced skin and organ lesions. There was also a lower occurrence of high-grade lesions and squamous cell carcinoma compared to animals fed a standard diet. These findings suggest that *Pennisetum glaucum* exerts hypoglycemic, antioxidant, and anti-inflammatory effects and could be considered a functional food with chemopreventive potential against HPV-related carcinogenesis. Future work should evaluate different millet concentrations and longer exposure periods.

## Figures and Tables

**Table 1:** Humane endpoint scores (arbitrary units) in the first and in the last weeks of the experiment (mean ± S.E.).

Group		1st Week	4th Week
1	WT, standard diet	0.00 ± 0.00	0.00 ± 0.00
2	WT, 36% *Pennisetum glaucum*	0.00 ± 0.00	0.00 ± 0.00
3	HPV+, standard diet	0.80 ± 0.20	1.20 ± 0.20
4	HPV+, 29% *Pennisetum glaucum*	0.80 ± 0.20	0.40 ± 0.24
5	HPV+, 36% *Pennisetum glaucum*	0.40 ± 0.24	0.60 ± 0.24

HPV—human papillomavirus. WT—wildtype. Statistically significant differences were not found (*p* > 0.05).

**Table 2:** Ponderal weight gain, body mass index, and murinometric parameters in all experimental groups (mean ± S.E.).

Group/Parameter	Group 1	Group 2	Group 3	Group 4	Group 5
	WT Standard Diet	WT 36% *P. glaucum*	HPV+ Standard Diet	HPV+ 29% *P. glaucum*	HPV+ 36% *P. glaucum*
Ponderal weight gain (%)	0.28 ± 3.10	−3.47 ± 2.18 *	21.46 ± 7.06	17.56 ± 9.42	4.30 ± 2.50
Body mass index	0.32 ± 0.01	0.33 ± 0.01	0.34 ± 0.01	0.38 ± 0.05	0.38 ± 0.02
Naso-anal length (cm)	10.50 ± 0.27	10.30 ± 0.12	9.90 ± 0.19	9.50 ± 0.45	9.60 ± 0.29
Naso-caudal length (cm)	19.50 ± 0.41	20.00 ± 0.20	19.10 ± 0.24	20.50 ± 1.16	19.10 ± 0.24
Abdominal perimeter (cm)	7.70 ± 0.20	7.70 ± 0.12	7.10 ± 0.19	7.70 ± 0.20	7.50 ± 0.16
Lee index	0.56 ± 0.01	0.57 ± 0.01	0.58 ± 0.01	0.62 ± 0.04	0.61 ± 0.02

*P. glaucum*—*Pennisetum glaucum*; WT—wildtype. * Statistically significant difference from group 3 (*p* < 0.05).

**Table 3:** Absolute (g) and relative organ weights (mean ± S.E.).

Group		Heart	Lungs	Liver	Spleen	Right Kidney	Left Kidney
1	WT, standard diet	0.18 ± 0.01 0.005 ± 0.000	0.23 ± 0.02 0.007 ± 0.001	1.45 ± 0.06 0.042 ± 0.001	0.21 ± 0.04 0.006 ± 0.003	0.26 ± 0.01 ^b^ 0.007 ± 0.000 ^c^	0.25 ± 0.02 0.007 ± 0.001
2	WT, 36% *P. glaucum*	0.41 ± 0.23 0.012 ± 0.007	0.21 ± 0.01 0.006 ± 0.001	1.39 ± 0.06 0.040 ± 0.003 ^a^	0.17 ± 0.05 0.005 ± 0.003	0.25 ± 0.01 0.007 ± 0.001	0.25 ± 0.01 0.007 ± 0.000
3	HPV+, standard diet	0.16 ± 0.01 0.005 ± 0.000	0.19 ± 0.01 0.006 ± 0.000	1.48 ± 0.04 0.044 ± 0.002	0.15 ± 0.02 0.004 ± 0.001	0.21 ± 0.01 ^d^ 0.006 ± 0.001	0.22 ± 0.01 0.007 ± 0.000
4	HPV+, 29% *P. glaucum*	0.15 ± 0.01 0.004 ± 0.000	0.20 ±0.01 0.006 ± 0.000	1.37 ± 0.05 0.041 ± 0.001	0.16 ± 0.01 0.005 ± 0.001	0.21 ± 0.01 ^d^ 0.006 ± 0.000	0.21 ± 0.01 0.006 ± 0.000
5	HPV+, 36% *P. glaucum*	0.17 ± 0.01 0.005 ± 0.000	0.21 ± 0.01 0.006 ± 0.001	1.52 ± 0.04 0.044 ± 0.002	0.18 ± 0.01 0.005 ± 0.001	0.25 ± 0.01 0.007 ± 0.000	0.24 ± 0.01 0.007 ± 0.000

*P. glaucum*—*Pennisetum glaucum*; WT—wildtype. ^a^ Statistically different from groups 3 and 5; ^b^ statistically different from groups 3 and 4; ^c^ statistically different from group 4; ^d^ statistically different from group 5.

**Table 4:** Hematological parameters, serum total proteins, and glucose (mean ± S.E.).

Group	Group 1	Group 2	Group 3	Group 4	Group 5
	WT Standard Diet	WT 36% *P. glaucum*	HPV+ Standard Diet	HPV+ 29% *P. glaucum*	HPV+ 36% *P. glaucum*
Red blood cell parameters					
Erythrocytes (M/μL)	9.40 ± 0.32	9.49 ± 0.39	10.01 ± 0.26	8.68 ± 0.56	8.58 ± 1.45
Hematocrit (%)	41.25 ± 3.30	39.60 ± 2.19	43.20 ± 1.04	41.20 ± 1.25	42.10 ± 2.13
Hemoglobin (g/dL)	14.24 ± 1.94	13.92 ± 0.70	14.82 ± 0.27	13.34 ± 0.62	13.58 ± 0.75
MCV (fL)	53.30 ± 6.49	49.50 ± 2.76	46.86 ± 1.01	46.04 ± 0.61	47.78 ± 1.58
MCH (pg)	15.92 ± 1.00	15.38 ± 0.40	14.80 ± 0.24	14.66 ± 0.17	15.14 ± 0.36
MCHC (g/dL)	30.05 ± 1.78	31.16 ± 1.57	31.60 ± 0.35	31.86 ± 0.42	31.70 ± 0.27
Reticulocytes (K/μL)	581.16 ± 442.36	584.14 ± 580.34	407.64 ± 45.78	399.54 ± 47.77	374.44 ± 49.90
White blood cell parameters					
Leukocytes (K/μL)	1.30 ± 0.54	1.96 ± 1.57	1.94 ± 3.15	0.90 ± 0.31	2.52 ± 1.85
Neutrophils (K/μL)	0.63 ± 0.55	0.79 ± 1.12	1.25 ± 2.23	0.21 ± 0.08	1.40 ± 1.78
Lymphocytes (K/μL)	0.64 ± 0.12	1.11 ± 0.88	0.76 ± 0.74	0.64 ± 0.24	1.03 ± 0.58
Monocytes (K/μL)	0.02 ± 0.01	0.04 ± 0.04	0.06 ± 0.09	0.03 ± 0.01	0.05 ± 0.02
Eosinophils (K/μL)	0.0075 ± 0.005	0.014 ± 0.009	0.012 ± 0.02	0.008 ± 0.004	0.022 ± 0.019
Basophils (K/μL)	0.0075± 0.005	0.004 ± 0.005	0.010 ± 0.012	0.010 ± 0.012	0.008 ± 0.008
Platelet parameters					
Platelets (K/μL)	1108.75 ± 128.32	1130.00 ± 117.24	1025.80 ± 78.24	940.00 ± 53.94	1061.40 ± 102.14
MPV (fL)	7.45 ± 0.24	7.62 ± 0.19	7.42 ± 0.16	7.58 ± 0.04	7.70 ± 0.19
PDW (fL)	6.95 ± 0.37	6.90 ± 0.38	6.78 ± 0.13	6.90 ± 0.23	7.00 ± 0.30
Serum parameters					
Total proteins (g/dL)	5.50 ± 0.42	5.44 ± 0.46	5.76 ± 0.30	5.28 ± 0.30	5.94 ± 0.33
Glucose (mg/dL)	163.75 ± 36.39	184.20 ± 10.10	243.40 ± 13.84 ^a^	207.20 ± 11.80 ^a^	178.00 ± 21.17

MCH—mean corpuscular hemoglobin; MCHC—mean corpuscular hemoglobin concentration; MCV—mean corpuscular volume; MPV—mean platelet volume; PDW—platelet distribution width; *P. glaucum*—*Pennisetum glaucum*; WT—wildtype. ^a^ Statistically different from groups 1, 2, and 5 (*p* < 0.05).

**Table 5:** Incidence of skin lesions in each experimental group, number (%).

Group (n = 5)		Group 1	Group 2	Group 3	Group 4	Group 5
		WT Standard Diet	WT 36% *P. glaucum*	HPV+ Standard Diet	HPV+ 29% *P. glaucum*	HPV+ 36% *P. glaucum*
Ear skin	Normal	5 (100%)	5 (100%)	0 (0%)	0 (0%)	0 (0%)
	Hyperplasia	0 (0%)	0 (0%)	0 (0%)	3 (60%)	4 (80%)
	Dysplasia	0 (0%)	0 (0%)	4 (80%)	2 (40%)	1 (20%)
	SCC	0 (0%)	0 (0%)	1 (20%)	0 (0%)	0 (0%)
Chest skin	Normal	5 (100%)	5 (100%)	0 (0%)	0 (0%)	0 (0%)
	Hyperplasia	0 (0%)	0 (0%)	3 (60%)	5 (100%)	5 (100%)
	Dysplasia	0 (0%)	0 (0%)	2 (40%)	0 (0%)	0 (0%)
	SCC	0 (0%)	0 (0%)	0 (0%)	0 (0%)	0 (0%)

*P. glaucum*—*Pennisetum glaucum*; SCC—squamous cell carcinoma; WT—wildtype.

**Table 6:** Incidence of lesions in the liver and spleen in each experimental group, number (%).

Group (n = 5)		Group 1	Group 2	Group 3	Group 4	Group 5
		WT Standard Diet	WT 36% *P. glaucum*	HPV+ Standard Diet	HPV+ 29% *P. glaucum*	HPV+ 36% *P. glaucum*
Liver	Normal	5 (100%)	5 (100%)	0 (0%)	1 (20%)	1 (20%)
	Grade I hepatitis	0 (0%)	0 (0%)	5 (100%)	4 (80%)	4 (80%)
	Grade II hepatitis	0 (0%)	0 (0%)	0 (0%)	0 (0%)	0 (0%)
Spleen	Normal	5 (100%)	5 (100%)	4 (80%)	2 (40%)	5 (100%)
	White pulp hyperplasia	0 (0%)	0 (0%)	1 (20%)	3 (60%)	0 (0%)

## Data Availability

The original contributions presented in this study are included in the article/Supplementary Materials. Further inquiries can be directed to the corresponding author.

## Supplementary Materials

The following supporting information can be downloaded at https://www.mdpi.com/article/10.3390/cimb47100858/s1.

## Abbreviations

The following abbreviations are used in this manuscript:

ANOVA	Analysis of variance
BW	Body weight
CAT	Catalase
E	Early
EDTA	Ethylene diamine tetraacetic acid
FELASA	Federation for Laboratory Animal Science Associations
GPx	Glutathione peroxidase
GR	Glutathione reductase
GSH	Glutathione in the reduced state
GSSG	Glutathione in the oxidized state
GST	Glutathione S-transferase
HPV	Human papillomavirus
IARC	International Agency for Research on Cancer
L	Late
LCR	Long control region
*P. glaucum*	*Pennisetum glaucum*
ROS	Oxygen reactive species
S.E.	Standard error
SOD	Superoxide dismutase
TBARS	Thiobarbituric acid reactive substances
WT	Wildtype
