# Supplementary material for: Pennisetum glaucum (L.) Oral Supplementation Mitigates Multi-Organic Dysfunction Associated with Carcinogenesis in HPV16-Transgenic Mice
Source: Curr Issues Mol Biol. 2025 Oct 17;47(10):858. doi: 10.3390/cimb47100858 (PMC12563015; doi:10.3390/cimb47100858)
Supplement: Supplementary file 1 [file cimb-47-00858-s001.zip › cimb-3858130-supplementary.pdf]

Phytochemical analysis of *Pearl millet* extract

HPLC-DAD Analysis of Phenolic Compounds

The phenolic profile of *Pearl millet* extract (5 mg/mL) was analyzed using high-performance liquid chromatography (HPLC) coupled with a diode array detector (DAD) from Thermo Electron (San Jose, CA, USA), integrated with a Gilson system (Villers-le-Bel, France). Separation was carried out using a C18 column (250 mm × 4.6 mm, 5 µm; ACE, Aberdeen, Scotland). The mobile phase consisted of solvent A (0.1% trifluoroacetic acid in water) and solvent B (0.1% TFA in acetonitrile), at a flow rate of 1 mL/min. Chromatograms were recorded at 254, 320, and 370 nm. Identification of phenolic compounds was based on comparisons of retention times, UV spectra, and maximum absorbance wavelengths with those of known standards (Iyda *et al.* 2019) (Figure S1).

Figure S1: Quantification of soluble and insoluble and condensed phenolics.

Quantification of Polyphenols

Total polyphenol content was determined using the Folin-Ciocalteu assay as described by Silva *et al*. Briefly, 20 µL of *Pearl millet* extract was mixed with 20 µL of distilled water and 100 µL of Folin-Ciocalteu reagent (diluted 1:10 in double-distilled water). After thorough mixing, 80 µL of 7.5% sodium carbonate (Na_2CO_3) was added. The reaction mixture was incubated in a water bath at 45°C for 15 minutes. Absorbance was then measured at 750 nm. Results were expressed as milligrams of gallic acid equivalents (mg GAE/g dry weight). Phenolic compounds were extracted from 200 g of *Pearl millet* flour using 5 mL of 70% methanol. The mixture was incubated

at 70°C for 30 minutes. After centrifugation, the supernatant (containing soluble phenolics) was separated, and the pellet was subjected to sequential extraction with 10 mL of 70% ethanol, followed by 100% ethanol, and finally 100% acetone. Each extraction step lasted 1 hour at room temperature. Supernatants were discarded. The remaining pellet was treated with 5 mL of trifluoroacetic acid (TFA) in 50% methanol and incubated at 80°C for 2 hours. Following this, 2 mL of the solution was collected and centrifuged at 13,000 rpm for 10 minutes to obtain the insoluble polyphenols. These fractions were analyzed using HPLC and the Folin-Ciocalteu method (Aires *et al*. 2020) (Figure S2).

Quantification of Tannins

Tannin content was determined according to Sumi *et al*. (2021), including both condensed and hydrolysable forms. Tannins were extracted by mixing 40 mg of millet flour with 950 µL of 70% methanol. The mixture was incubated at 70°C for 30 minutes. The supernatant was then filtered using a 0.2 µm Spartan filter and stored in amber vials to prevent light-induced degradation (Sumi *et al*. 2021). To quantify condensed tannins, 20 µL of extract was added to 100 µL of vanillin reagent (4% vanillin in methanol) and 50 µL of concentrated hydrochloric acid (HCl). The mixture was incubated in the dark for 20 minutes, and absorbance was recorded at 500 nm. Results were expressed as mg catechin equivalents per gram of dry weight (mg CAE/g DW). For hydrolysable tannins, 50 µL of extract was mixed with 150 µL of 2.5% (w/v) potassium iodate. The solution was kept in the dark for 15 minutes, and absorbance was measured at 550 nm. Tannic acid was used as the standard, and results were expressed as mg tannic acid equivalents per gram of dry weight (mg TAE/g DW) (Dewanto *et al*. 2002) (Figure S2).

Quantification of Flavonoids

Total flavonoid content was assessed using the aluminum chloride ($AlCl_3$) colorimetric method according to Dewanto *et al*. (2002). In summary, 25 µL of PM extract was combined with 100 µL of ultra-pure water and 10 µL of 5% sodium nitrite ($NaNO_2$). After 5 minutes of incubation in the dark at room temperature, 15 µL of 10% $AlCl_3$ was added. Following a further 6-minute dark incubation, 100 µL of 1 M sodium hydroxide (NaOH) and 50 µL of ultra-pure water were added, and the mixture was mixed again. Absorbance was measured at 510 nm. Results were expressed as milligrams of catechin equivalents (mg CAE/g dry weight) (Figure S2).

Figure S2: Hydrolysable and condensed polyphenols, tannins and flavonoids.

Non enzymatic antioxidant activity: Anti-DPPH

The evaluation of the antiradical capacity of the extract was described by Gorinstein *et al*. (2004). This method is based on the ability of antioxidants to neutralize the 2,2-diphenyl-1-picrylhydrazyl (DPPH) radical by donating a hydrogen atom, thereby converting it into a hydrazine form. The antiradical activity is quantified by measuring the reduction in DPPH absorbance. Initially deep violet in color, DPPH turns yellowish upon reaction with antioxidant compounds. To perform this assay, 200 µL of the plant extract at various concentrations was mixed with 2800 µL of a methanolic DPPH solution (0.1 mM). The mixture was vigorously shaken for 30 seconds and then incubated in the dark at room temperature for 30 minutes. Absorbance was measured at 517 nm using a blank without extract as the reference (Siddhuraju and Becker, 2003) (Figure S3). The antiradical activity was expressed as the percentage of inhibition of free DPPH radicals compared to a control methanolic DPPH solution, which was considered 100%. The inhibition percentage was calculated using the following formula :

$$\%\ \text{inhibition} = [(Abs_{100}\% - Abs\_sample) / Abs_{100}\%] \times 100$$

Figure S3: % 2,2-diphenyl-1-picrylhydrazyl (DPPH) radical inhibition.

References

Aires A, Carvalho R. Kiwi fruit residues from industry processing: study for a maximum phenolic recovery yield. J Food Sci Technol. 2020 Nov;57(11):4265–76.

Dewanto V, Wu X, Adom KK, Liu RH. Thermal processing enhances the nutritional value of tomatoes by increasing total antioxidant activity. J Agric Food Chem. 2002 May;50(10):3010–4.

Iyda JH, Fernandes Â, Ferreira FD, Alves MJ, Pires TCSP, Barros L, et al. Chemical composition and bioactive properties of the wild edible plant Raphanus raphanistrum L. Food Res Int. 2019 Jul;121:714–22.

Perumal Siddhuraju, Klaus Becker (2003). Antioxidant properties of various solvent extracts of total phenolic constituents from three different agroclimatic origins of drumstick tree (Moringa oleifera Lam.) leaves. *Journal of Agricultural and Food Chemistry*, *51.*, 2144–2155.

Sumi SA, Siraj MA, Hossain A, Mia MS, Afrin S, Rahman MM. Pharmacological Activities of Ficus racemosa and Analysis of Its Major Bioactive Polyphenols by HPLC-DAD. Highlights on Medicine and Medical Science Vol 5. 2021 Jun;68–84.
